# Incarcerated transmesosigmoid hernia presenting in a 60-year-old man: a case report

**DOI:** 10.1186/1752-1947-2-161

**Published:** 2008-05-17

**Authors:** Danielle Collins, Dara Kavanagh, Eddie Myers, Steve Richards, Enda McDermott

**Affiliations:** 1 Surgical Professorial Unit, St. Vincent's University Hospital, Elm Park, Dublin 4, Ireland

## Abstract

**Introduction:**

Internal hernias are a rare cause of small bowel obstruction and are estimated to account for 1% to 5% of cases. Herniation through a defect in the sigmoid mesocolon constitutes 6% of all internal hernias.

**Case presentation:**

In this case report we describe a rare case of a fit and healthy 60-year-old man, with no previous history of abdominal surgery, who presented with signs and symptoms of small bowel obstruction as a result of an incarcerated transmesosigmoid hernia. The hernia was reduced and the incarcerated loop of small bowel was found to be viable. The patient made a good recovery and was discharged home on the fourth post-operative day.

**Conclusion:**

Internal hernias can cause considerable morbidity and mortality, so prompt diagnosis is paramount. Transmesosigmoid hernias are most common in the paediatric population; however, our patient was 60 years old. This report highlights the importance of considering an internal hernia as a cause of small bowel obstruction in individuals of all age groups and especially in those without a previous history of abdominal surgery.

## Introduction

Small bowel obstruction is a frequent surgical emergency. The most common causes of small bowel obstruction are post-operative adhesions and abdominal wall hernias [1].

In patients without a previous history of abdominal surgery other, less-common causes should be considered. Internal hernias are an infrequent cause of small bowel obstruction and are estimated to account for 1% to 6% of all cases.

## Case presentation

**Figure 1:**
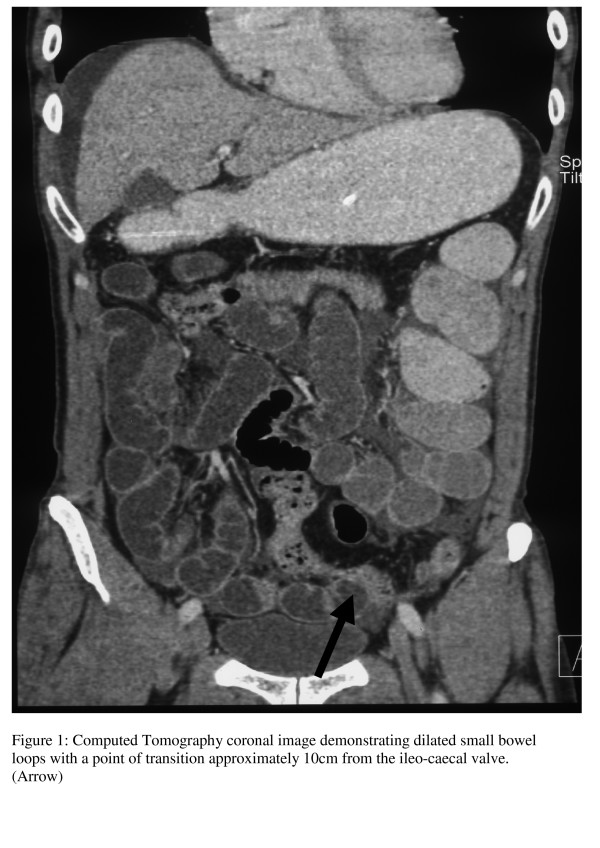
**Coronal CT demonstrating dilated small bowel with a transition point 10 cm from the ileocaecal valve.** (Arrow).

A 60-year-old man presented acutely with a 1-day history of colicky central abdominal pain, abdominal distension, vomiting and absolute constipation. He had no previous history of abdominal surgery. Physical examination revealed dry mucous membranes and tenderness in the right lower quadrant. Bowel sounds were increased and digital rectal examination was normal. Laboratory investigations revealed a leucocytosis of 11.4×10^9^g/dl. Plain abdominal radiography demonstrated two prominent loops of small bowel in the right lower quadrant. He was initially managed conservatively. A Ryle's nasogastric tube was inserted and he was resuscitated with intravenous fluids. A computed tomography (CT) scan of his abdomen and pelvis (Figure 1) demonstrated dilated loops of small bowel with a zone of transition approximately 10 cm from the ileo-caecal valve. Distally, the small bowel was collapsed. After 24 hours of conservative therapy his clinical picture remained unchanged. Based on his clinical and CT findings a decision was made to perform an exploratory laparotomy.

**Figure 2:**
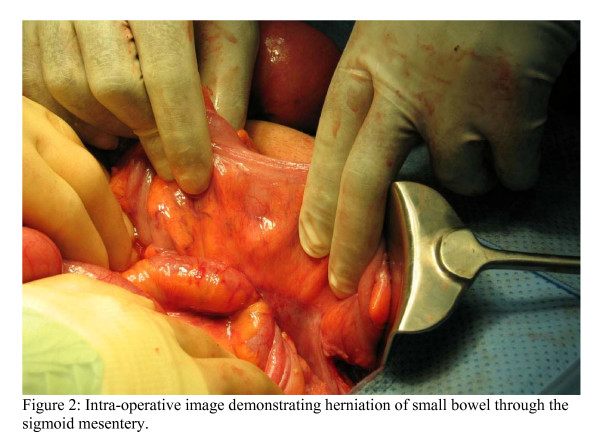
Intraoperative image demonstrating herniation of small bowel through the sigmoid mesentery.

**Figure 3:**
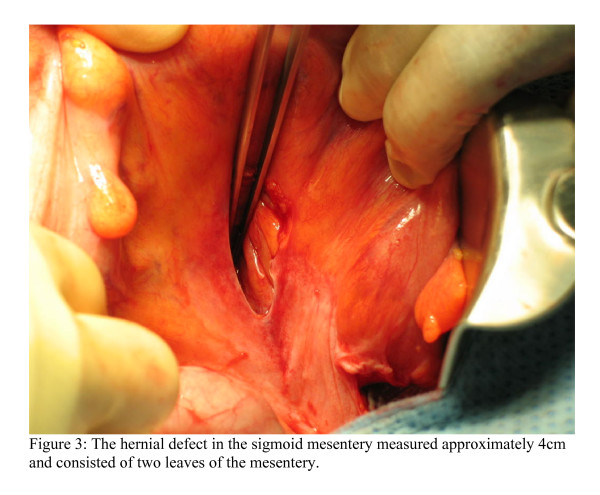
The hernial defect measured approximately 4 cm and consisted of two leaves of sigmoid mesentery.

At laparotomy, a mechanical small bowel obstruction due to an incarcerated internal hernia was found. A loop of ileum had herniated through a congenital defect in the sigmoid mesocolon (Figure 2). This was reduced successfully and the defect was approximated with interrupted 3/0 poliglecaprone sutures. The strangulated portion of small bowel was viable. In this case the hernial orifice measured 4 cm and consisted of two leaves of the sigmoid mesentery (Figure 3). No hernial sac was present.

## Discussion

An internal hernia is the protrusion of a viscus through a normal or abnormal peritoneal or mesenteric aperture within the abdominal cavity. It is estimated to account for 0.6% to 5.8% of small bowel obstructions with transmesosigmoid hernias constituting 6% of all internal hernias [2].

Previously, paraduodenal hernias were regarded as the most common type of internal hernia; however, it has recently been reported that transmesenteric hernias are increasing in incidence [3]. Transmesenteric hernias are a recognised complication of laparoscopic (Roux-en-Y) bariatric surgery and liver transplantation when surgically created mesenteric defects are not closed [4]. Although transmesenteric hernias are increasing in incidence, transmesosigmoid herniation is very rare, especially in patients with no previous history of abdominal surgery or trauma.

Congenital internal hernias of the sigmoid mesentery are divided into three categories: intersigmoid, intramesosigmoid and transmesosigmoid [5]. Transmesosigmoid hernias occur when a loop of small bowel passes through a defect in the sigmoid mesentery. This type of hernia involves the two layers of the mesentery and does not have a hernial sac. The underlying embryology of this defect has not been fully elucidated; however, there are several theories as to how these defects arise. It has been suggested that the mesenteric defect is due to partial regression of the dorsal mesentery or due to inadequate vascularisation of the enlarging mesentery during foetal development [6]. Alternatively the mesentery can be torn following abdominal trauma [7].

In cases of transmesosigmoid hernias, patients tend to present acutely with abdominal pain and signs of small bowel obstruction [8,9]. However, making a pre-operative diagnosis of an internal hernia can be difficult. Radiological investigations such as CT of the abdomen or a small bowel contrast study can be invaluable [10]. CT has a reported sensitivity of 63% and a specificity of 73% in diagnosing internal hernias. In a review of 17 cases of internal hernia [11], it was found that an abnormal cluster of small bowel loops outside the colon with central displacement of the colon and engorgement of mesenteric vessels were suggestive of trans-mesenteric hernias. In addition, the lack of omental fat covering these small bowel loops raises a suspicion of an internal hernia.

The management of internal hernias requires reduction of the hernia and repair of the defect by either a laparoscopic or open approach. In these cases there is a high incidence of small bowel ischaemia and infarction and resection of the strangulated small bowel segment may be necessary. Iatrogenic internal hernias can be successfully managed by laparoscopy [12] and laparoscopic repair of congenital internal hernias has been described [9]. In our case, a laparotomy was performed due to the uncertainty of a pre-operative diagnosis.

## Conclusion

This case highlights a rare cause of intestinal obstruction in a patient with no previous abdominal surgery. Interestingly, congenital internal hernias are more common in the paediatric population [6]. The current case was a 60-year-old man. The developmental evolution of the mesenteric defect has not been fully elucidated. There is a high incidence of strangulation and small bowel ischaemia with subsequent morbidity and mortality. Nowadays, with the widespread use of laparoscopic abdominal surgery, internal hernias may become an increasing surgical problem. In cases of small bowel obstruction without previous abdominal surgery, a congenital internal hernia should be considered. Prompt diagnosis and intervention enabling active management are paramount.

## Competing interests

The authors declare that they have no competing interests.

## Authors' contributions

DC drafted the article, performed the literature search and acquired the radiology images. DK assisted in performing the surgery, acquired the intraoperative images and reviewed the manuscript. EM supervised and edited the manuscript. SR and EMcD performed the surgery. All authors have read and approved the final manuscript.

## Consent

Written informed consent was obtained from the patient for publication of this case report and any accompanying images. A copy of the written consent is available for review by the Editor-in-Chief of this journal.
